# Evaluation of anti‐acne activity of human whole saliva against acne vulgaris: An in vitro study

**DOI:** 10.1111/jocd.16369

**Published:** 2024-11-09

**Authors:** Tyween Jia Coutinho, Suneel Dodamani, Atrey J. Pai Khot, Vaibhav Kumar, Sultan A. Almalki, Inderjit M. Gowdar

**Affiliations:** ^1^ Jawaharlal Nehru Medical College, KLE Academy of Higher Education and Research Belagavi Karnataka India; ^2^ Dr. Prabhakar Kore Basic Science Research Centre, KLE Academy of Higher Education and Research Belagavi Karnataka India; ^3^ Department of Public Health Dentistry, Faculty of Dental Sciences King George's Medical University Lucknow Uttar Pradesh India; ^4^ Department of Public Health Dentistry, Dr. GD Pol Foundation YMT Dental College and Hospital Navi Mumbai Maharashtra India; ^5^ Department of Preventive Dental Sciences, College of Dentistry, Prince Sattam bin Abdul Aziz University Alkharj 11942 Kingdom of Saudi Arabia; ^6^ Present address: Department of Public Health Dentistry, Goa Dental College and Hospital Bambolim‐Goa India

**Keywords:** acne vulgaris, antimicrobial, whole human saliva

## Abstract

**Background:**

Acne vulgaris (AV) is a multifactorial inflammatory skin disorder, affecting 9.45% of the world's population. AV can be painful, discomforting, and disfiguring due to scarring, leading to physiological distress, and economic burden. AV pathogenies can be due to various factors, key ones include follicular plugging, colonization by the microorganism, endocrinological factors, and inflammation. Gram‐positive bacteria *Cutibacterium acnes* and *Staphylococcus epidermidis* are the most common pathogens isolated from patients with AV.

**Aim:**

The present study aimed to investigate the anti‐acne activity of whole human saliva against bacteria that cause AV.

**Materials and Methods:**

The saliva sample from four different individuals was collected at three different intervals. These samples were used to perform antimicrobial susceptibility test (AST) against the pathogens. Kirby–Bauer disk diffusion susceptibility test, and Broth dilution method using Resazurin were performed.

**Results:**

The human saliva was effective in inhibiting *Cutibacterium acnes* and *Staphylococcus epidermidis* those causing AV. The Minimum inhibitory activity and disc diffusion assay depicted potency of saliva. The pH of the saliva samples was found to be more acidic in the morning samples when compared with the evening samples. The afternoon sample was found to be more effective when compared with the other two intervals.

**Conclusions:**

Saliva shows effective anti‐acne activity by inhibiting the growth and proliferation of acne‐causing bacteria.

## 1 INTRODUCTION

**FIGURE 1:**
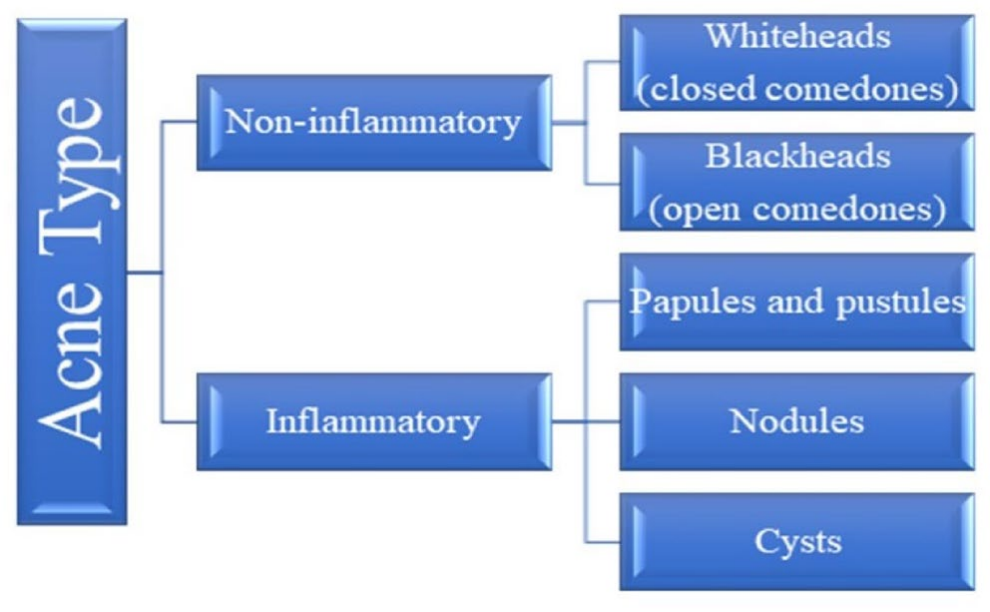
Classification of acne vulgaris.

**FIGURE 2:**
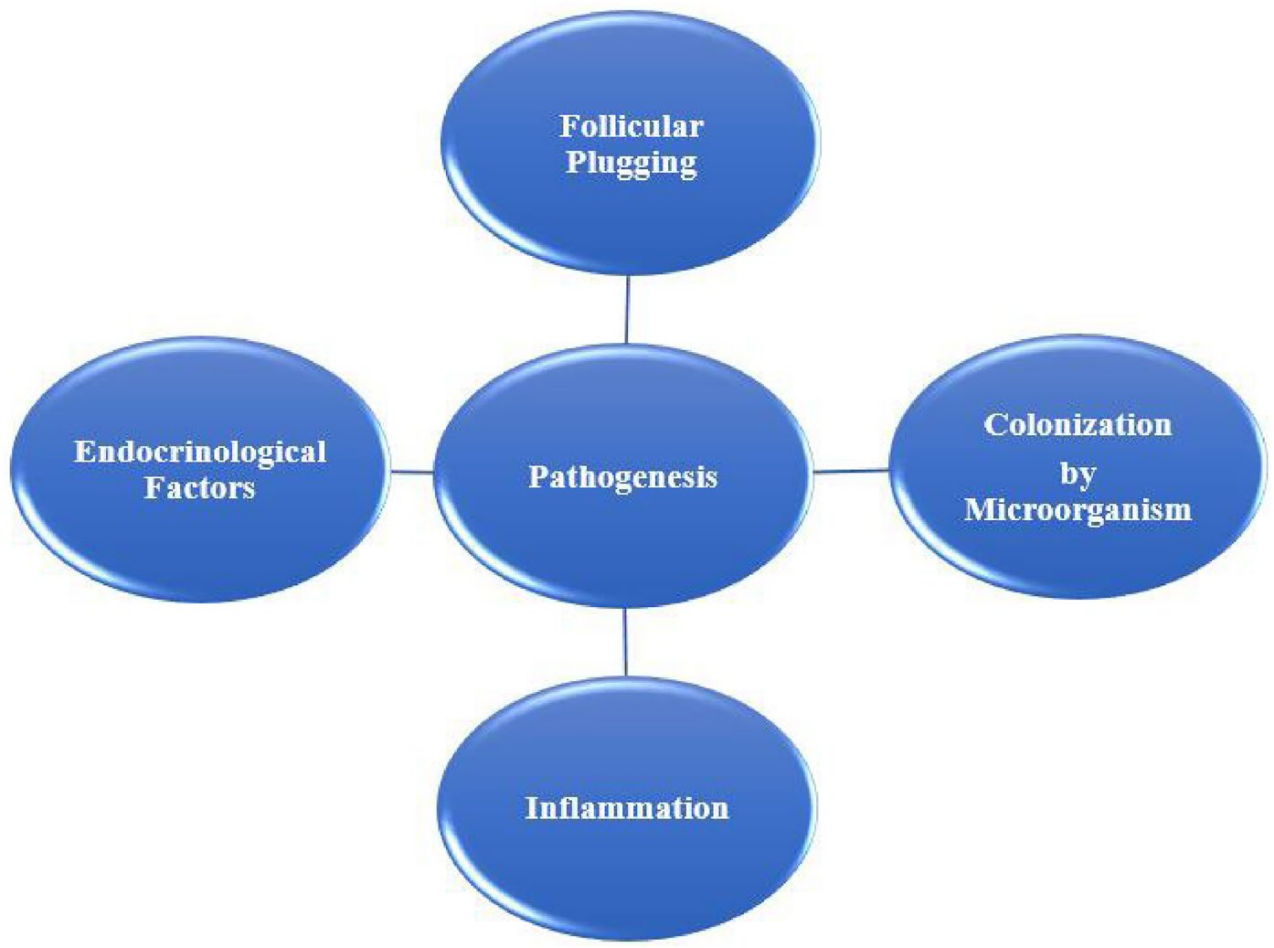
Key factors of acne pathogenesis.

Acne vulgaris (AV) is an epidemic of chronic, multifactorial inflammatory skin disorders.^1^ Characterized by the formation of comedones, papules, pustules, nodules, and cysts, which are the consequence of inflammation and blockage of the hair follicles and their associated sebaceous gland. Present as inflammatory lesions, noninflammatory lesions, or a mixture of both, primarily affecting the face and the upper trunk.^2^ Current research from around the globe has put forth the view that the frequency of individuals affected by AV is largely consistent globally. In the next half‐decade, individuals affected by AV in India are estimated to reach 23 million at a compound average growth rate of 0.5%. Even though AV is a skin disease, it can cause major emotional and psychological problems.^3^ Though not a lethal or debilitating disorder, AV can be painful, discomforting, and disfiguring due to scarring, leading to physiological distress, and economic burden.^4^ Acne can be classified into two categories: non‐inflammatory and inflammatory (Figure 1). AV pathogenesis is multifactorial, with its key factors being follicular plugging, colonization by the microorganism, endocrinological factors, and inflammation (Figure 2). The bacterial colonization of Gram‐positive bacteria *Cutibacterium acnes*, and *Staphylococcus epidermidis* are the most studied and common pathogens isolated from patients with AV.^5^

The treatment of AV is directed towards the known pathogenesis, which includes follicular excess sebum, *C. acnes*, hyperproliferation, and inflammation. Treatment is aimed at lessening the severity and reappearance of skin lesions, leading to a tweaked appearance. Acne is treated using topical or systemic antibiotics, topical retinoids, benzoyl peroxide, hormones, physical therapy using laser phototherapy, and chemical peels (Table 1).^6^, ^7^ When an individual is treated with antibiotics, there is a chance of developing resistant bacteria.^2^ To overcome these resistant strains, newer natural and traditional methods of treatment are in demand.

**TABLE 1:** A summary of currently available treatment approaches for acne.

Sr. no.	Type of therapy	Agent/approach	Activity
1	Topical therapy	Topical antibiotics	Inhibit growth of bacteria, and reduce inflation
		Topical retinoids (tretinoin, adapalene, and tazarotene)	Target abnormal follicular epithelial hyperproliferation, reduce follicular plugging, and reduce microcomedones
		Benzoyl peroxide	Anti‐inflammatory, keratolytic, and comedolytic activity
		Other topical agents	Used individually or in combination bactericidal, comedolytic, and anti‐inflammatory activity
2	Systemic therapy	Systemic antibiotics	Oral antibiotics for moderate‐to‐severe inflammatory acne
		Hormonal therapy	Anti‐androgen therapy‐prevention of androgen action on sebaceous gland, and follicular keratinocytes
		Isotretinoin	Effects all causative mechanisms of ace: changes abnormal follicular keratinization, decreases sebum production by 70%, decreases *C. acnes* colonization, and inflammations
3	Physical therapy	Lesion removal	Comedone extraction, chemical peels, and microdermabrasion, intra‐lesion corticosteroid injection for acne cysts
		Phototherapy	High‐intensity, narrow‐band blue light or blue, and red light: also, excision, and suturing, and laser resurfacing for scars

The present study aimed to investigate the anti‐acne activity of whole human saliva against bacteria that cause AV. The human whole saliva was used in the present study mainly because of its physiological and antioxidant properties, as it is a complex mixture of some useful secretary molecules like proline‐rich proteins, mucins, secretory immunoglobulin A, lysozyme, lactoferrin, and amylase.^21^ Anecdote: use of saliva to treat wounds and pimples is very common in Indian households. It is also observed in the animal kingdom that, when wounded, they are seen licking their wounds. Saliva has been used in diagnostics for more than 2000 years.^22^ Therefore, we have selected whole human saliva as one of the potential anti‐acne agents, and hence we have evaluated various anti‐acne activities.

## 2 MATERIALS AND METHODS

### 2.1 Microbial strain and culture condition

The bacteria like *Cutibacterium acnes* (formerly *Proprionibacterium acnes*) and *Staphylococcus epidermidis* were obtained from Microbial Type Culture Collection and Gene Bank, Chandigarh (MTCC 1951), and National Centre for Cell Science (NCCS), Pune (MCC 2044) respectively. *C. acnes* was revived using Rabbit blood agar and *S. epidermidis* on Nutrient agar.

### 2.2 Saliva sample collection and composition

In the present experiment, non‐stimulated saliva samples were collected by aspiration into 5 mL graduated centrifuge tubes. The collected saliva was filtered through 0.25 mm Nylon filter tubes to remove most of the microorganisms, and pH analysis was undertaken. The saliva samples were collected from all the six participants. However, participants with previous antibiotic intake over a period of 1 month were excluded from the study making the final sample size of four participants with a 3:1 female–male ratio belonging to the age group of 20–25 years. Written informed consent was obtained from the participants for the sample collection. They were designated as Sample (**A**), Sample (**B)**, Sample (**C**), and Sample (**D**). Morning saliva collection was done by the participants before brushing their teeth, between 7:30 a.m. and 8:00 a.m., with no oral intake of solid or liquid food. Afternoon saliva was collected at 1:00 p.m. before having lunch, and evening Saliva was collected at 5:00 p.m. The pH was determined using pH paper [HiMedia Pvt. Ltd., India].

### 2.3 Antimicrobial susceptibility test

#### 2.3.1 Broth dilution assay using resazurin method

In 96 well culture plates, after selecting specific wells, 100 μL of Brain Heart Infusion Broth [Himedia, M210I] was added. Further, 100 μL of human saliva [as 100%] was added. After this, a serial dilution was carried out up to five concentrations (50%, 25%, 12.5%, 6.25%, and 3.12%). Next, 10 μL of bacterial inoculums [0.5 Mc Farlands constant of 1.5 × 10^8^ CFU/mL] was added in each well, except for positive control. The plates were incubated for 24 h at 37°C. Further, 20 μL of resazurin (5 mg/10 mL distilled water) (HiMedia Pvt. Ltd, Mumbai, India) solution was added to each well and incubated for 1–4 h at 37°C. Recorded the colour change concentration at which resazurin was reduced to resorufin by a colour change from blue to ‘light pink’ which was taken as the Minimum Inhibitory Concentration.

#### 2.3.2 Kirby–Bauer disk diffusion susceptibility test

Prepared petri plates containing Nutrient Agar [NA] [Himedia, Pvt Ltd, Mumbai India] and the overnight grown bacterial broth [1.5 × 10^8^ CFU/mL] were diluted using 0.90% Phosphate buffered saline [PBS] to attain a turbidity of 0.5 McFarland standards. Further, seed 10 μL of 0.5 McFarland bacterial broth onto the solidified NA plates using a sterile cotton swab and let it dry for 2 min. Using sterile forceps, place a 6‐mm sterile disc [Himedia Pvt. Ltd., Mumbai, India] onto the seeded plates. Around 10 μL of centrifuged saliva sample was added onto the disk. The plates were then incubated at 37°C for 36 h.

## 3 RESULTS

The aseptically collected saliva is evaluated for antimicrobial activity and how it can help in the treatment of acne. Saliva samples were collected at three different intervals and were subjected to Antimicrobial Suspensibility Testing (AST). The saliva samples were collected at three different intervals—morning before brushing and consumption of any oral drinks or foods, in the afternoon before lunch, and in the evening. The morning saliva pH was in the acidic range of 5.5–6.5. Afternoon saliva pH ranged from acidic to neutral 6–7, and evening pH was 6.5–7. The pH of the samples, as reported in Table 2, morning saliva was the most acidic among the other two intervals, and the evening samples were close to neutral.

**TABLE 2:** Average pH of the saliva samples.

Sample	Time interval	pH
A	Morning	5.5
	Afternoon	6
	Evening	6
B	Morning	5.5
	Afternoon	6
	Evening	6.5
C	Morning	6
	Afternoon	6.6
	Evening	7
D	Morning	6.5
	Afternoon	7
	Evening	7.5

**FIGURE 3:**
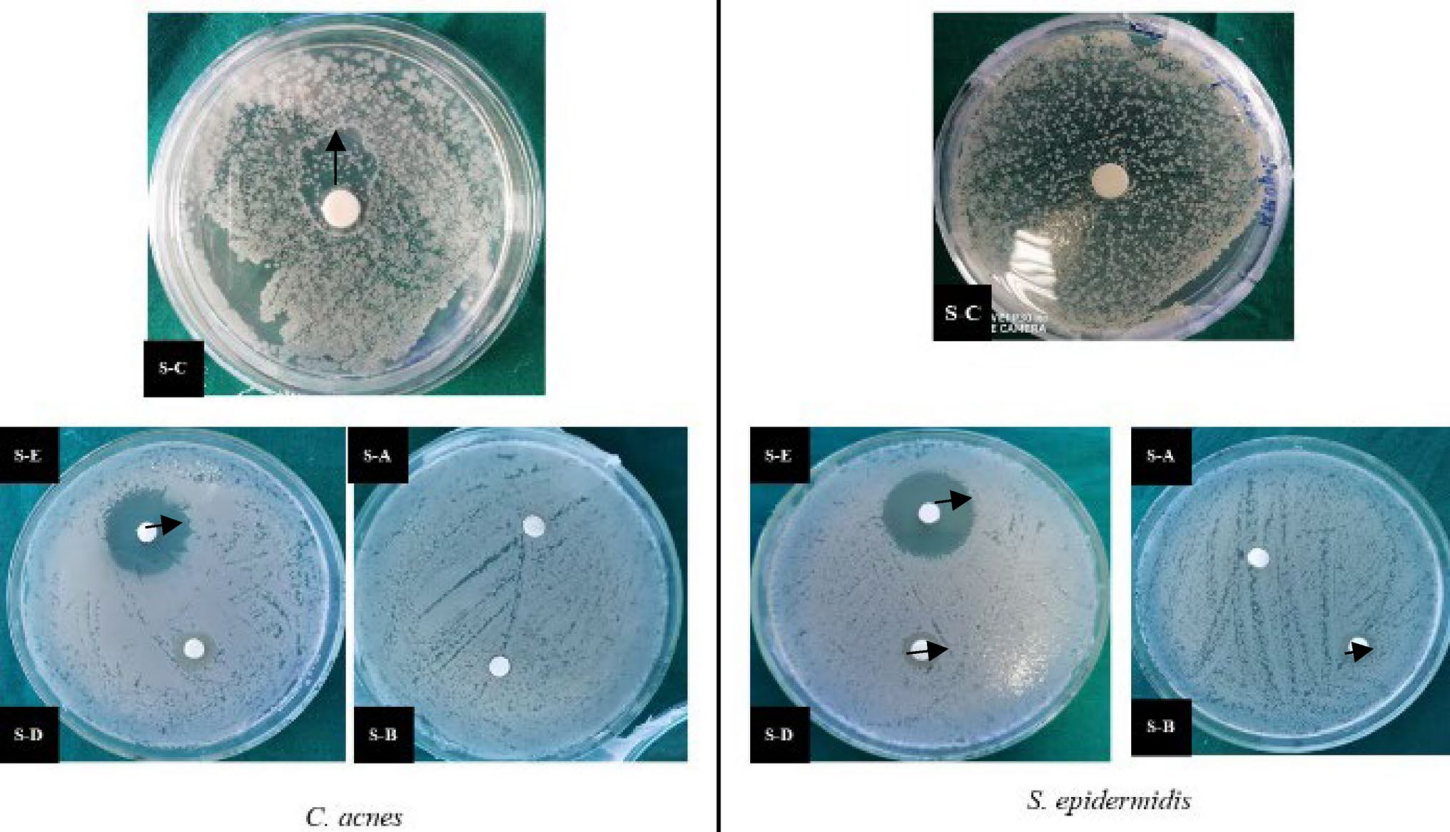
Kirby–Bauer disk diffusion susceptibility test using morning saliva sample against *Cutibacterium acnes* and *Staphylococcus epidermidis*; S‐A—Sample A; S‐B—Sample B; S‐C—Sample C; S‐D—Sample D; and S‐E—erythromycin. Arrow mark indicates zone of inhibition.

**FIGURE 4:**
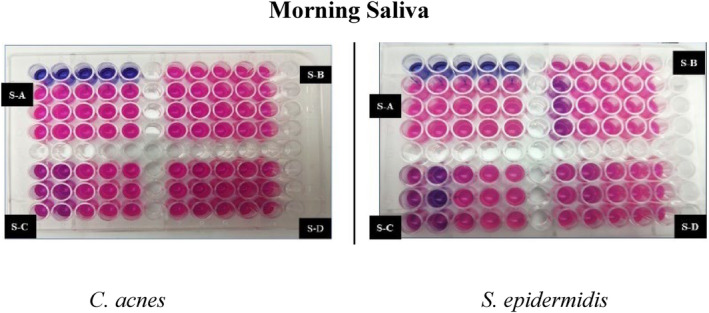
Broth dilution method using resazurin, morning saliva samples against *Cutibacterium acnes* and *Staphylococcus epidermidis*.

**FIGURE 5:**
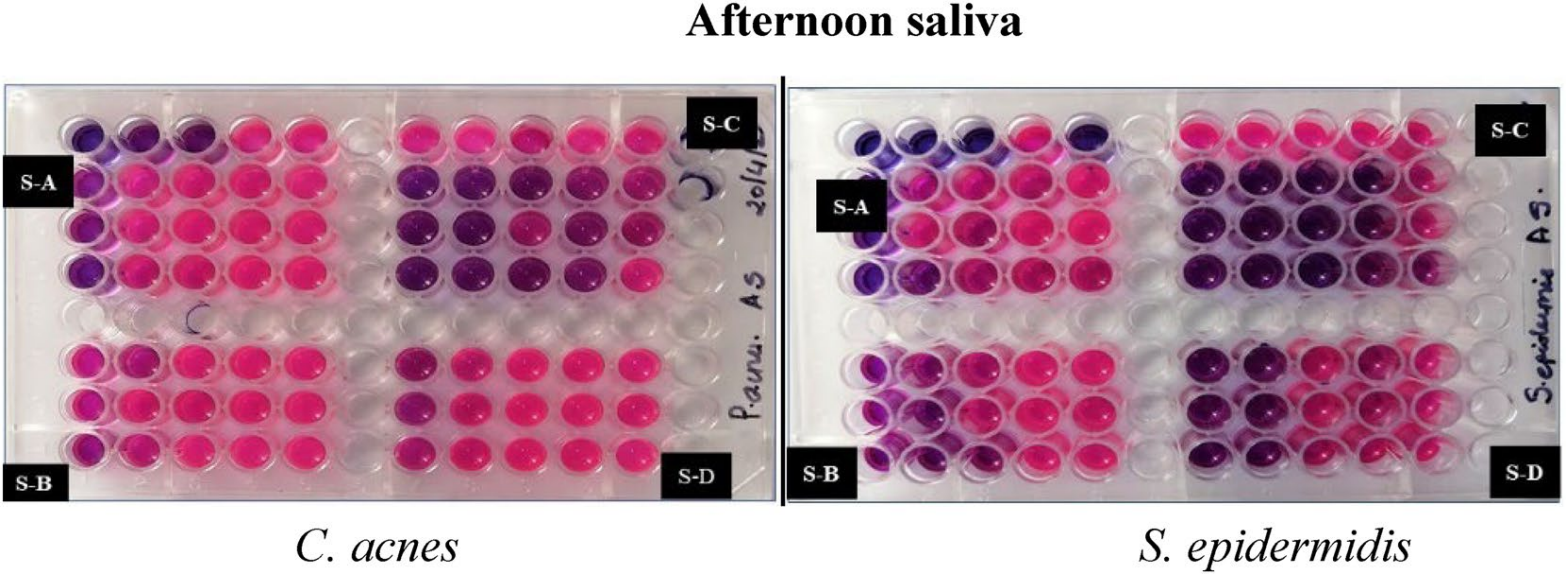
Broth dilution method using resazurin, afternoon saliva samples against *Cutibacterium acnes* and *Staphylococcus epidermidis*.

**FIGURE 6:**
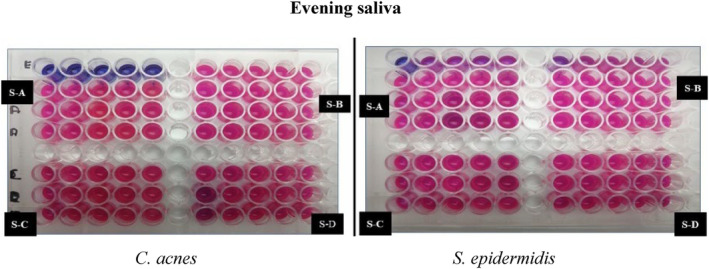
Broth dilution method using resazurin, evening saliva samples against *Cutibacterium acnes* and *Staphylococcus epidermidis*.

Kirby–Bauer disk diffusion susceptibility test was done. As seen in Figure 3 the zone of inhibition was observed in samples B, C, and D. Similarly, positive and very effective results were seen in the broth dilution method (Figures 4, 5, 6).

## 4 DISCUSSION

AV is the most common disorder of the human skin, afflicting up to 80% of individuals during their lifetime.^23^ In the treatment of AV, topical antibiotics such as clindamycin, erythromycin, and tetracycline are mostly used. However, resistance to these antibiotics when used in monotherapy has drastically increased over the past few decades. This has led to the search for and development of newer and more natural methods for treating AV. In the present study, an attempt was made to investigate the effectiveness of whole human saliva against the growth of acne‐causing bacteria. *C. acnes* is a Gram‐positive anaerobic bacterium, that colonizes the sebaceous glands and plays a major role in the pathogenies of AV,^5^ and Gram‐positive *Staphylococcus epidermidis* is also responsible for acne development.^8^ The whole human saliva was tested against the two bacteria.

In this study, afternoon saliva proved to be more effective when compared with the other two intervals. Further, saliva supports interactions among the oral microbiome, host innate immune cells, and their secreted immune modulators, and it is a transporter for salivary proteins, thus unifying host defenses against *Candida albicans*.^23^ From the results, it can be inferred that a higher concentration of the sample is highly effective in reducing bacterial aggregation. This reduction can be a result of the antimicrobial proteins that are present in the saliva.^9^ This included lactoferrin (Lf), lactoperoxidase (Lp), lysozyme (Lz), salivary peroxidase (Sp), myeloperoxidase, thiocyanate concentrations, and antimicrobial peptides.^10^ Narendra Maddu, 2019, reported that effectors such as Lz, Lf, and Lp with other components of saliva can have an effect on the growth and proliferation of the oral bacteria, either by acting as bactericidal or bacteriostatic.^11^ This can be a possible reason for the reduction in bacterial concentration when saliva was used against *C. acnes* and *S. epidermidis*. Further, during the sleep, the bacteria in the mouth continue to metabolize carbohydrates and produce acids as by‐products. With less saliva flow to wash away these acids, they can accumulate and lower the pH of saliva, ultimately saliva becomes more acidic in the morning.^24^

### 4.1 Lactoferrin

The Lf acts as an effective bacteriostatic molecule against iron consuming bacteria for their growth and activity, by competing with the iron chelation molecules.^11^ Lf is also able to prevent biofilm formation by sequestering iron ions that result in the twitching of bacteria. This reduction in biofilm formation will in turn increase the susceptibility of other antimicrobial molecules in saliva, as biofilm reduces the effect of antibiotics due to aggregation and increases bacterial resistance.^10^ Lf plays a role in inflammatory as well as non‐inflammatory immune responses. In the inflammatory responses, Lf acts as a chemoattractant that leads to the accumulation of neutrophil granulocytes at the bacterial infection site. The interleukin‐1β‐ and interleukin‐8‐mediated inflammation is tempered due to the binding of Lf, thereby controlling the host immune response.^12^

### 4.2 Lysozyme

Another antibacterial enzyme that is present in the saliva is Lz. Lz was the first discovered antibacterial protein that is present in mucosal fluids like tears, saliva, respiratory, and cervical secretions, acting as the first line of defense.^10^ Lz has a bactericidal action. The enzyme cleaves the peptidoglycan moiety, which is a mesh‐like layer made of sugar and amino acids that make up the cell walls of the bacteria, through the hydrolysis of b‐(1, 4) linkages between the N‐acetylmuramic acid (NAM) and N‐acetylglucosamine (NAG) saccharides.^13^ This catalytic activity of the enzyme affects the cell wall integrity, leading to the lysis of the bacterial cell.

### 4.3 Salivary peroxidase

The two main salivary peroxidases are lactoperoxidase and myeloperoxidase. This enzyme catalyzes the oxidation of salivary thiocyanate by hydrogen peroxide to hypothiocyanite and hypothiocyanous acids. Hypothiocyanite is known to have antimicrobial activity, as it affects bacterial metabolic activity by causing the inactivation of hexokinase, a sulfhydryl group of the enzymes that are involved in glycolysis and the transport of carbohydrates in a bacterial cell.^14^ Secretory IgA increases the activity of the enzyme.^11^

### 4.4 Antimicrobial peptides

APs are the most important salivary components involved in defense against microflora. Examples of AP are the defensins, histatins, and cathelicidins LL‐37.^9^ These cationic peptides bind to the negatively charged bacterial cell wall. This binding leads to transmembrane pore formation, causing a lethal efflux of vital cell constituents.^15^, ^16^

All the above‐mentioned are a few of the salivary components that exhibit antimicrobial activity. These proteins can be further studied to develop antibiotic agents that can be used to treat AV, as the already marketed products have severe side effects and can lead to resistance in microflora due to overuse.

Saliva also exhibits wound healing properties due to the presence of naturally occurring growth factors such as epidermal growth factor, vascular endothelial growth factor, transforming growth factor‐alpha, transforming growth factor‐beta, nerve growth factor, fibroblast growth factor, and insulin‐like growth factor.^17^ It is also seen that histatins enhance the healing of wounds through the re‐epithelialization process and promote endothelial cell adhesion.^18^ This property of saliva can be explored, as it will help to develop products that help with healing the scarring that occurs in inflammatory cysts and severe acne.

With previous observation, when saliva was applied topically, there was a reduction in sebum in the acne. One of the possible reasons for this could be the activity of the lipase enzyme that is present in the saliva.^19^ The enzyme is responsible for fat digestion from foods in the oral cavity. The sebum consists of fatty acids/lipids, mainly triglycerides, wax esters, and squalene.^20^ The topical application might lead to the breakdown of these lipid constituents of the sebum, which acts as a source of nutrients for the proliferation of the pathogens, thereby limiting bacterial growth. It was also observed that the inflammation in the acne was reduced when the saliva was applied to the papules and pustules, as Lf, along with its antibiotic properties, has anti‐inflammatory properties.^11^

Further studies are required to assess the ability of saliva to inhibit the formation of biofilm, which increases the resistance of the bacteria against the test antibiotic. Individuals' assessment of the salivary antimicrobial proteins and peptides against acne‐causing bacteria needs further investigation. An in vivo investigation would also help in determining the effectiveness of saliva as a treatment for AV.

## 5 LIMITATIONS OF THE STUDY

The present study used a limited number of saliva samples, an extensive study could be done with more inclusion and exclusion criteria, for example, history of oral antibiotics used for acne treatment, and a broader age group consisting adolescents to young adults. More experiments regarding the reduction of biofilm need to be conducted, as it is one of the crucial targets for treatment. A further study to determine the salivary components and their concentrations at different time intervals could help provide a better understanding.

## 6 CONCLUSION

The inhibitory test using whole human saliva at different intervals showed effective anti‐bacterial activity. The test sample, when used for Kirby–Bauer Disk Diffusion and Broth dilution methods, gave notable results against *C. acnes* and *S. epidermidis*, which are the most studied bacteria responsible for the pathogenesis of AV. Various components of saliva have been known to be effective in the treatment of microbial infection, for example., Lf, Lz, Lp, and APs mainly histatin. Saliva is also known to have wound healing and anti‐inflammatory activity, which can possibly help in the reduction of scarring papule and pustule formation. It was also observed that there was a difference in the biofilm formed in wells with a higher concentration of saliva.

## Acknowledgement

The authors extend their appreciation to KAHER’s Dr. Prabhakar Kore Basic Science Research Center (BSRC), Belagavi for providing resources for the study.

## AUTHOR CONTRIBUTIONS

**Tyween Jia Coutinho**: Conceptualization, Design, Data collection & processing, Literature search, Writing—original draft. **Suneel Dodamani**: Methodology, revising intellectual content, data curation, supervision, and final approval. **Atrey J. Pai Khot**: Conceptualization, validation, investigation, data curation, manuscript editing, and manuscript review. **Vaibhav Kumar**: Formal analysis, revising intellectual content, data curation, critical review, and editing. **Sultan A. Almalki**: Resources, revising intellectual content, funding acquisition, supervision and final approval. **Inderjit M. Gowdar**: Resources, revising intellectual content, funding acquisition, supervision and final approval.

## FUNDING INFORMATION

Prince Sattam bin Abdulaziz University funded this research work through the project number (PSAU/2024/01/88903).

## CONFLICT OF INTEREST STATEMENT

There was no conflict of interest associated with this original research article.

## ETHICS STATEMENT

Ethical clearance was obtained from Institutional Ethical and Review Committee. The study was done in accordance with World Medical Association Declaration of Helsinki.

## Data Availability

The data that support the findings of this study are available from the corresponding author upon reasonable request.
